# Low Energy Pulsed Laser Excitation in UV Enhances the Gas Sensing Capacity of Photoluminescent ZnO Nanohybrids

**DOI:** 10.3390/s19245490

**Published:** 2019-12-12

**Authors:** Argyro Klini, Maria Androulidaki, Demetrios Anglos

**Affiliations:** 1 Institute of Electronic Structure and Laser, Foundation for Research and Technology-Hellas, P.O. Box 1385, GR 71110 Heraklion, Crete, Greece; pyrhnas@physics.uoc.gr (M.A.); anglos@uoc.gr (D.A.); 2 Department of Chemistry, University of Crete, GR 71003 Heraklion, Crete, Greece

**Keywords:** ZnO nanoparticles, ZnO-PDMS nanocomposite, ZnO photoluminescence, gas sensing, optical sensor, ethanol sensing, oxygen sensing

## Abstract

Nanohybrids, composed of luminescent zinc oxide (ZnO) nanoparticles dispersed in an inert polydimethylsiloxane (PDMS) matrix, exhibit an excellent ability to follow changes in the type and composition of their surrounding atmosphere. These changes are found to affect the UV photoluminescence (PL) emission of the ZnO-PDMS hybrids measured at room temperature. The influence of irradiation parameters, such as excitation intensity and wavelength, on the response of the ZnO-PDMS sensor against ethanol and oxygen, have been systematically investigated in a comparative study performed employing pulsed excitation at 248 and 355 nm. This study represents the first demonstration that the sensing performance of the PL-based ZnO sensors can be optimized by tuning the excitation parameters and it particularly illustrates that maintaining a low pump energy density is crucial for enhancing the sensitivity of the sensor achieving response values approaching 100%.

## 1. Introduction 

Zinc oxide and, in particular, various nanostructures of it have been actively studied in the context of chemical sensing science and technology [1]. To date, sensing by zinc oxide (ZnO) has been achieved almost entirely by monitoring variations in the electrical conductance of properly prepared nanostructured substrates of the semiconductor exposed in varying environments. This approach has enabled quantitative detection of various volatile compounds [2] including hydrocarbons [3] alcohols or small gaseous oxides [4] with appreciable detection limits, often in the low ppm (part per million) range [5], as well as analysis of biological compounds in solutions [6].

However, a considerable drawback of conductivity-based sensing stems from the fact that ZnO nanostructures achieve efficient performance as sensors only at elevated temperature, typically in the range of 200–400 °C [7], and this questions their potential use in the presence of flammable gases or explosives and for the analysis of biomaterials. With the objective to overcome this barrier and improve the sensitivity of the chemoresistive sensors, a number of studies have been undertaken, showing that photoactivation of the sensing material via irradiation in the ultraviolet at room temperature [8] lead to improved sensitivity. In one such study [9], it was demonstrated that the optimal light intensity, required for photoactivation, is analyte dependent, hence irradiation dose could be used as a means for tuning the selectivity of the sensor with respect to a specific target analyte. Despite this promising outlook, the response level of such UV activated sensors remains considerably lower in comparison to that of the thermally activated ones [10,11]. 

Monitoring the optical properties of semiconductor materials [12,13,14], such as transmittance, reflectance, or photoluminescence (PL) emission, which have also been found to undergo changes in the presence of external chemical stimuli, appears a promising alternative for gas sensing albeit such an approach is much less investigated, to date. Importantly, changes in optical properties are observable at room temperature, and their recording requires straightforward experimental equipment, while the sensing material itself does not require any elaborate preparation for a measurement to be performed. These features, as well as the capability of remote monitoring, make optical sensing an attractive technology for achieving fast and reliable gas detection even in hostile environments. 

In this context, optical sensors based on the photoluminescence of ZnO nanostructures have been studied for the detection of different compounds such as NO_2_ [15,16], CO [17], H_2_ [18], ethanol [19], or oxygen [20]. Recently, Liu et al. [21] demonstrated excellent optical sensing properties of ZnO-CuO heterostructured nanorods towards H_2_S at room temperature, achieving sub-ppm sensitivity. Concerning larger molecules in solution, glucose sensing was recently demonstrated [22] based on the quenching of photoluminescence from ZnO nanorods deposited on a gallium nitride (GaN) substrate, with sensitivity of 1.4 %/mM over a wide range of glucose concentration (0.5–30 mM). 

Most of the studies reported exploit the UV exciton emission of ZnO and its dependence on the surrounding environment, employing excitation in the near UV, normally provided by a continuous wave (cw) laser or a lamp [23] with photon energy exceeding the band gap of the oxide. However, pulsed excitation was also found to work quite well as reported in a recent study in which monitoring of ethanol in air [19] was achieved via probing spectral changes in the PL emission of ZnO excited by the third harmonic of a nanosecond-pulsed Nd:YAG, laser at 355 nm. Measurements performed with ZnO nanoparticles dispersed in a polymeric matrix (polydimethylsiloxane, PDMS) exposed to ethanol concentration as low as 0.4 Torr (equivalent to 520 ppm), showed a response, on the order of 4%, significantly higher compared to 1.5% recorded when ZnO nanowires exposed to 1000 ppm of ethanol were excited by a cw laser (He-Cd laser at 325 nm) [24]. An added advantage of pulsed laser excitation stems from the ability to record the emission in a time-gated mode, thus minimizing effects of stray light, particularly important for standoff and remote detection applications.

The mode of excitation, pulsed or cw, may be equally important from the mechanistic point of view, considering the influence of pump rate on the population of photogenerated electrons and holes [17] forming in ZnO upon excitation. The density of carriers plays a decisive role in the sensing process by affecting the kinetics of redox reactions occurring between the analyte gas and the semiconductor surface. [25]. In the present work a comparative study is reported, for the first time, investigating the response of ZnO-PDMS composites exposed to ethanol and oxygen, as a function of photoexcitation parameters with emphasis on the excitation wavelength, *λ_exc_* (photon energy), and the energy fluence, *F_exc_* (photon number density). The aim is to optimize the potential of the PL-based sensing methodology in ZnO nanostructures and understand the physical limitations, in an effort to enhance the sensor performance and determine a regime for optimal operating conditions. 

## 2. Experimental Methods

All measurements in the present study made use of a well-established nanohybrid system composed of ZnO dispersed in PDMS [19]. Nanocomposites containing 40% w/w ZnO (6% v/v) were prepared by mixing the appropriate amount of commercially available ZnO nanoparticles (in powder form), having an average particle size 125 ± 25 nm and 99.9% purity (Aldrich 20, 553-2), with silanol-terminated poly(dimethyl)siloxane PDMS (PS348.7:PS343.5 = 5:1) and tetraethyl orthosilicate (TEOS), as detailed elsewhere [26]. 

Laser-induced photoluminescence measurements were performed with the ZnO-PDMS samples placed inside a custom built optical cell that, via several ports and valves, permitted variation and control of the ambient atmosphere [19]. A commercial ethanol probe (PS-2194, Pasco) was attached via a special port and used for monitoring changes in ethanol vapor pressure during measurements. A Pirani gauge (Thermovac TM20, Leybolt Hereous, Radeburg, Germany) was used for measuring the background pressure. Excitation was provided by laser pulses at 355 nm or 248 nm incident normally onto the sample surface, with energy density (*F_exc_*) in the range of 0.05−4.5 mJ/cm^2^. Pulses at 248 nm, with time width of 15 ns, were delivered from a KrF excimer laser (LPX 200, Lambda-Physik, Göttingen, Germany). Excitation at 355 nm, with pulse width of 8 ns, came from the third harmonic output of a flash-lamp pumped Q-switched Nd:YAG laser (SL404, Spectron Laser Systems, Edinburgh, UK). The PL emission was collected approximately at 45° with respect to the sample normal, by means of a multimode fused silica optical fiber coupled into the entrance slit of a spectrograph (01-002AD, PTI, USA) equipped with a 300 lines/mm holographic grating. Emission spectra were recorded on an intensified charge coupled device detector (ICCD, DH740-18F-03, Andor iStar, Andor Technology Belfast, UK). Typically, spectra were averaged over 50 laser pulses with the laser operating at a repetition rate of 8 Hz. The photoluminescence emitted by the ZnO-PDMS hybrids and its variation as a function of changes in the ambient atmosphere was monitored continuously by acquiring spectra at 10 s intervals and calculating the integrated emission intensity, I = ∫I(λ)dλ, in the spectral range of 370–500 nm. To correct for any effects arising from fluctuations of the excitation laser beam intensity, the integrated PL intensity was normalized with respect to that of the laser line, scattered off of the sample surface, which was also recorded in the spectra, albeit highly attenuated, thanks to the low but non-vanishing transmission of a long-pass filter used in front of the spectrograph entrance slit. 

## 3. Results and Discussion

### 3.1. Ethanol Sensing

**Figure 1:**
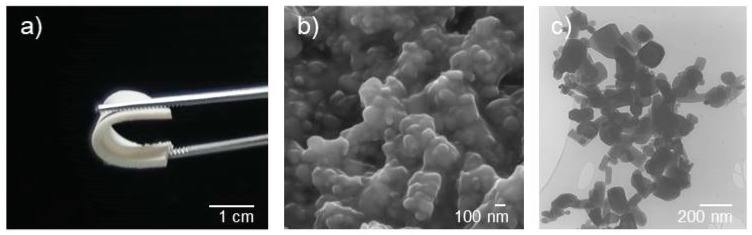
Optical (**a**), SEM (**b**), and TEM (**c**) images of ZnO-PDMS nanohybrid containing 40% w/w ZnO.

The ZnO-PDMS nanohybrids consist of luminescent zinc oxide (ZnO) nanoparticles dispersed in the soft, flexible, and optically inert PDMS matrix which offers a high degree of processability in view of potential applications [26]. A macroscopic view of a typical nanocomposite sample is shown in Figure 1 along with SEM and TEM images of the nanoparticle dispersions.

**Figure 2:**
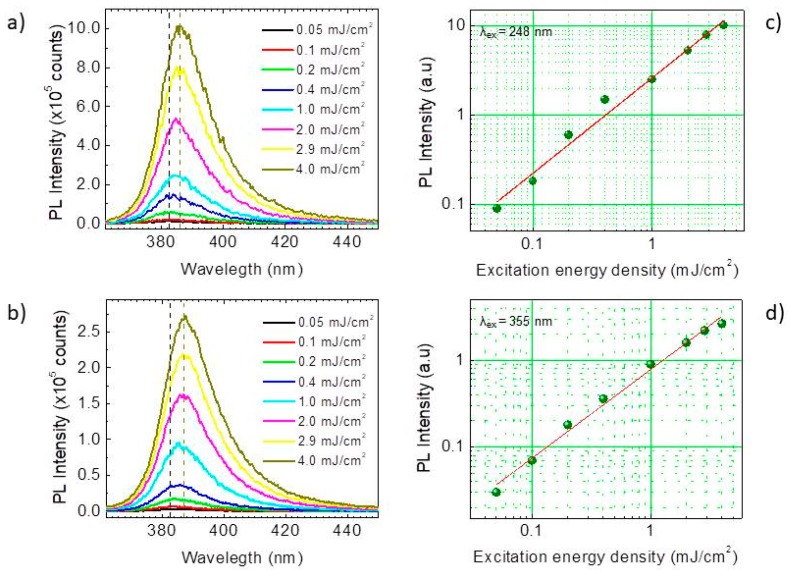
Room temperature photoluminescence (PL) emission spectra of ZnO-PDMS in air, upon excitation with (**a**) 248 nm and (**b**) 355 nm laser pulses as a function of energy density (*F_exc_*). Dependence of the PL emission intensity on *F_exc_* with excitation at (**c**) 248 nm and (**d**) 355 nm respectively. The solid lines in graphs c and d representlinear fits of the data, to equation: logI = A + B × log(F_exc_) with (**c**) A = 0.4, B = 1.07 and (**d**) A = 0.1, B = 1.02.

In order to assess how the photoluminescence emission of ZnO-PDMS depends on pump source and energy density, PL spectra were recorded at room temperature (RT, Τ = 293 Κ, ambient air), in a series of measurements involving excitation of the hybrids with ultra violet laser pulses (248 nm/5.0 eV and 355 nm/3.5 eV), at fluence values, ranging from 0.05 to 4.5 mJ/cm^2^ (peak power density on the order of 10^3^–10^5^ W/cm^2^). All spectra, as seen in Figure 2, show a strong emission centered at about 382 nm, characteristic of the ultraviolet ZnO photoluminescence, and any defect photoluminescence in the visible is negligible [19]. The UV emission arises from the recombination of photogenerated charge carriers across the band-gap of the semiconductor and in the examined excitation regime its intensity varies linearly (Figure 2c,d) with excitation fluence (*F_exc_*). The shape of the spectrum remains unaffected (FWHM ~18 nm) when pump intensity is varied, whereas its peak red shifts from 382 nm (3.246 eV) to 386 nm (3.212 eV). This is in good agreement with the red-shift of the electron-hole plasma (EHP) emission observed in bulk ZnO, a consequence of the band gap renormalization (BGR) induced by the carrier density increase [27]. 

**Figure 3:**
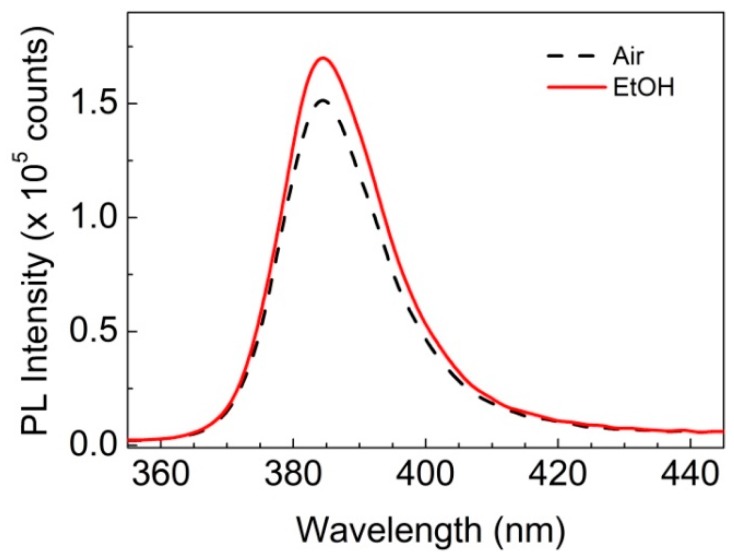
Room temperature PL emission spectra of 40% w/w ZnO-PDMS in air (dotted line) and in 0.5 Torr ethanol-enriched atmosphere (solid line) upon excitation with *λ_exc_* = 248 nm and *F_exc_* = 0.2 mJ/cm^2^.

In accordance with previous experimental observations [24] the ZnO-PDMS nanocomposites respond when exposed to ethanol vapors showing a distinct enhancement of their UV PL emission in comparison to that recorded in air (Figure 3). 

To investigate this response with regards to the ability of the ZnO-PDMS hybrids to follow changes in the ethanol content of the surrounding atmosphere, the PL emission was monitored over time for a number of successive cycles of air-ethanol exposure [19]. The relative change in the integrated PL emission intensity, %ΔI, defined as the sensor response, is calculated according to the following expression: $\%\Delta I=\frac{I_{EtOH}-I_{Air}}{I_{Air}}$100%, where *I_Air_* and *I_EtOH_* represent the integrated PL intensity of ZnO-PDMS in air and ethanol vapor atmosphere, respectively. 

**Figure 4:**
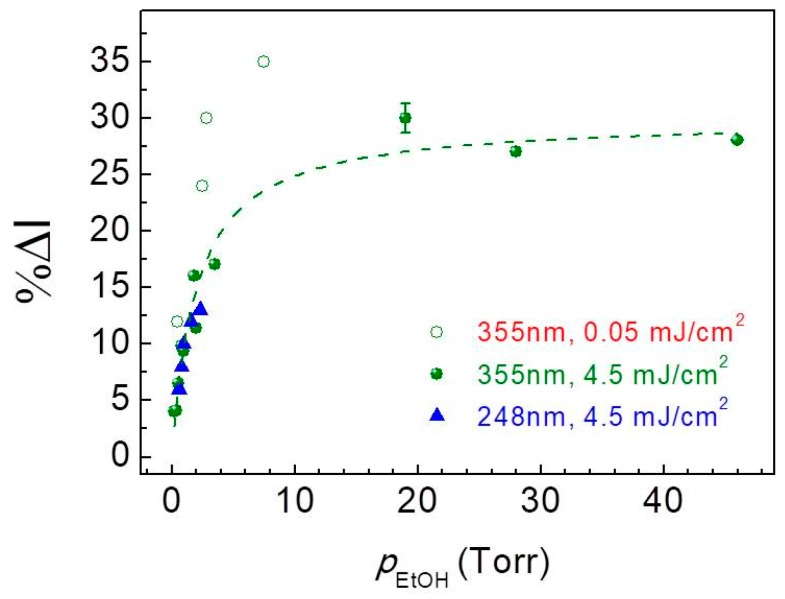
Relative change in the integrated PL emission intensity (%Δ*I*) as a function of ethanol vapor pressure upon excitation at 248 nm (triangles) and 355 nm (full circles) at *F_exc_* = 4.5 mJ/cm^2^. Open circle symbols represent %Δ*I* obtained with excitation *λ_exc_* = 248 nm and *F_exc_* = 0.05 mJ/cm^2^. The dotted line is a fit to a Langmuir-type function, %Δ*I* = A × *p_EtOH_*/(1 + B × *p_EtOH_*) with A = 14.9, B = 0.5 [19].

In our earlier study, it was shown that ZnO-PDMS composites, optically excited at 355 nm, allow the detection of ethanol in a wide vapor pressure range (*p_EtOH_*), from 50 down to 0.4 Torr [19]. At the low pressure regime, the sensor response, %Δ*I*, was found to exhibit a linear increase with ethanol vapor pressure with excitation at 355 nm or 248 nm, both at 4.5 mJ/cm^2^ of energy density (Figure 4). Moreover, the sensitivity [28] of the sensor for *p_EtOH_* < 2 Torr is similar, 7%/Torr, for both excitation wavelengths. This behavior implies similar photochemical activity on the semiconductor surface even though irradiation is provided by photons of different energy. In a similar case, reported in the literature, the response of a UV-activated, resistive ZnO nanofiber sensor to 20 ppm of ethanol (equivalent to 0.015 Torr) was found to be similar for photoactivation at either 365 nm or 254 nm [29]. 

**Figure 5:**
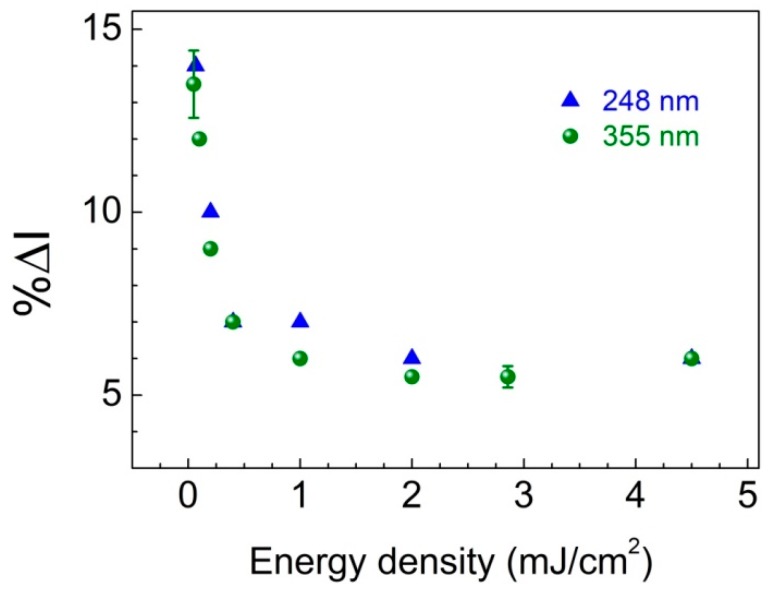
Relative change in the integrated PL emission intensity, %Δ*I*, as a function of excitation energy density with 248 and 355 nm laser pulses, following exposure to ethanol vapors with *p_EtOH_* = 0.6 Torr.

It is noteworthy that excitation at 355 nm with low energy density (see data in Figure 4, open circle symbols) appears to lead to considerably enhanced response of the sensor in comparison to high fluence excitation. Based on this observation, the dependence of ethanol detection response on excitation energy density was more systematically explored exposing the ZnO-PDMS hybrids to low ethanol vapor pressure atmosphere (*p_EtOH_* = 0.6 Torr), which is close to the limit of detection of our sensor. In Figure 5, %Δ*I* is plotted, as a function of *F_exc_* both for excitation at 248 nm and 355 nm. It is clearly evident that low excitation energy density favors the response of ethanol detection. On the other hand, increasing energy density leads to decrease of sensor response, which reaches a low plateau corresponding to about one third of the highest response. The onset of this low plateau (Figure 5) occurs at about 0.2 mJ/cm^2^ for the two wavelengths investigated and might be indicative either for a saturation behavior or for a change in the mechanism of interaction between the semiconductor and the analyte. 

The widely manifested increase of the ZnO PL emission upon exposure of the semiconductor to ethanol vapors has been interpreted on the basis of charge transfer occurring from the alcohol to ZnO that leads to an increase in the density of charge carriers, which contribute to the enhancement of radiative recombination following excitation of the semiconductor [25]. Even though the detailed chemistry, taking place on the semiconductor surface during the interaction with the analyte has not been clearly identified, a series of reactions of the photogenerated electrons and holes ($\left(ZnO\right)+hv\to e^{-}+h^{+}$) with the adsorbed molecules have been proposed to explain the PL sensing mechanism in metal oxides [23]. According to these models, at ambient temperature and atmosphere, oxygen molecules O_2_ are physically adsorbed on the surface of ZnO. Upon photoexcitation of the semiconductor, adsorbed O_2_ molecules are reduced to $O_{2}^{-}$ ions ($O_{2}+e^{-}\to O_{2}^{-}$) [9]. The extraction of electrons from the conduction band [12] leads to the formation of an electron depleted layer, the thickness of which has been suggested to have an impact on the UV photoluminescence leading to a reduction of its intensity [21]. When ethanol is present, it interacts with the photo-generated oxygen ions ($O_{2}^{-}$) [9] causing the release of trapped electrons back to ZnO [25], thereby reducing the depletion layer thickness and enhancing the ZnO PL emission over that observed in air. An alternative mechanism, suggesting a competing adsorption between the oxygen species and ethanol on the surface of ZnO nanostructures, has also been proposed, on the basis of a limited number of theoretical [30] and experimental [19] studies, to be responsible for ethanol sensing, owing to the ability of ethanol to remove oxygen from the semiconductor surface, while releasing back the electrons trapped by oxygen.

To this end, the effect of the high excitation density, *F_exc_*, leading to reduced sensing response, can be viewed as a consequence of the increased density of charge carriers, which influences the reactions of the photogenerated species (e.g., $O_{2}^{-}$) with ethanol altering the adsorption/desorption kinetics involved [31]. Of particular significance appears to be the value of *F_exc_*, where the response curve enters the low plateau regime (Figure 5), and this is found to correlate well with the Mott transition excitation density for ZnO. Indeed, considering that each absorbed photon of the excitation pulse creates one electron-hole pair and taking into account the absorption coefficient of ZnO (*α*_248_ = 3 × 10^5^ cm^−1^ and *α*_355_ = 2 × 10^5^ cm^−1^) [32], it is calculated that with *F_exc_* at about 0.3 and 0.2 mJ/cm^2^ (for 248 and 355 nm, respectively) the semiconductor reaches its Mott transition density (n_Mott_ = 1.5 × 10^18^ cm^−3^ at 300 K) and electron-hole plasma (EHP) is generated [33]. The enhanced spatial separation of charge carriers occurring at this stage alters dramatically the reaction rates and adsorption/desorption equilibrium of the analyte on the semiconductor surface.

### 3.2. Oxygen Sensing

**Figure 6:**
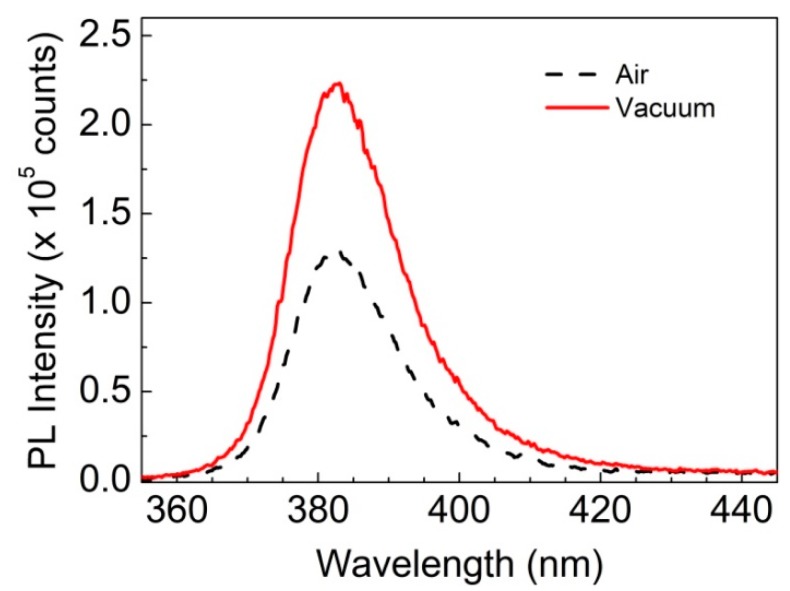
Room temperature PL emission spectra of 40% w/w ZnO-PDMS in air (dotted line) and in low vacuum (7.5 × 10^−2^ Torr) atmosphere (solid line) upon excitation with *λ_exc_* = 248 nm and *F_exc_* = 0.6 mJ/cm^2^.

The strong indication that oxygen plays an active role in the ethanol sensing process prompted a further investigation, which compared how the UV PL emission of ZnO might be affected by the presence or absence of O_2_. Indeed, a clear effect is noticed when PL spectra of the ZnO-PDMS hybrids (Figure 6) are measured at atmospheric pressure or in low vacuum (*p* = 7.5 × 10^−2^ Torr), consistently with previous findings [34] showing that the emission intensity of ZnO decreases significantly in the presence of oxygen, which is attributed to the formation of $O_{2}^{-}$ species on the semiconductor surface ($O_{2}+e^{-}\to O_{2}^{-}$).

**Figure 7:**
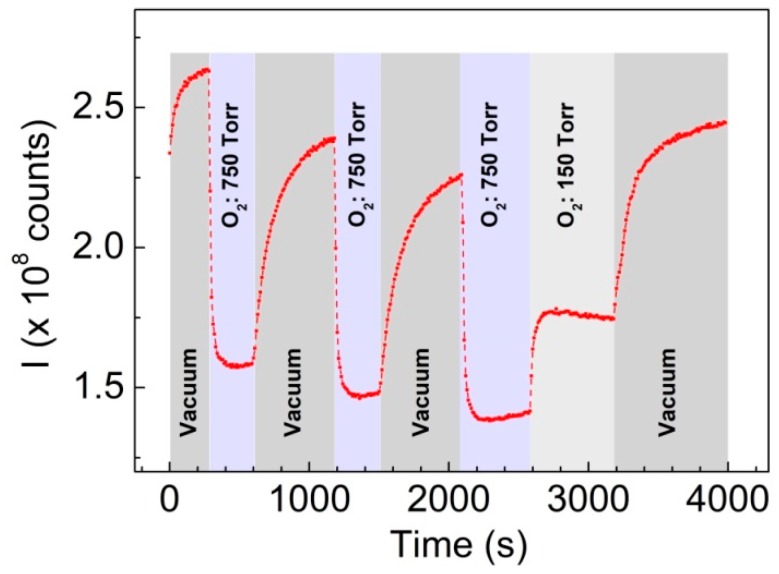
Room temperature PL emission (integrated intensity) following in time the exposure of ZnO-PDMS nanohybrids to successive cycles of different oxygen concentration environment (*λ_exc_* = 248 nm, *F_exc_* = 0.6 mJ/cm^2^).

Figure 7 shows a typical dynamic response curve of the sensor, under 248 nm excitation, illustrating the quenching/enhancement of PL emission due to the presence/absence of O_2_. The time-resolved measurements of the integrated emission intensity, *I*, upon exposure to different levels of oxygen pressure, ranging from 7.5 × 10^−2^ to 750 Torr, illustrate also that ZnO-PDMS hybrids can be effectively used for monitoring the environmental changes in oxygen ambient.

**Figure 8:**
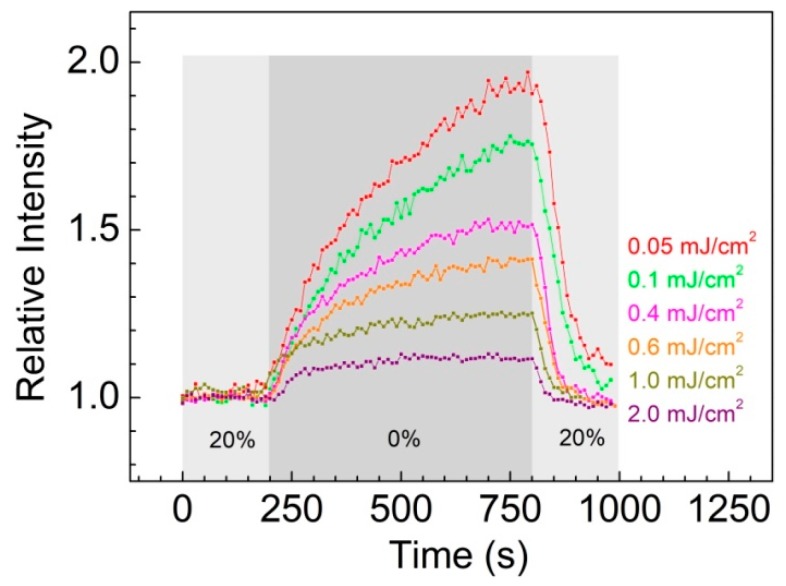
PL emission intensity, as a function of time following exposure of the ZnO-PDMS system to 20% and 0% O_2_ (low vacuum and atmospheric air) cycles, at *λ_exc_* = 248 nm and different *F_exc_*.

The dependence of the sensor response, %ΔI, on excitation energy density for both 355 nm and 248 nm excitation was recorded by exposing the ZnO-PDMS hybrids to various cycles interchanging low vacuum (0% O_2_) with atmospheric air (20% O_2_) conditions. Similarly to the case of ethanol sensing, excitation intensity was found to be a critical parameter affecting the response of the sensor. It is evident from the response traces shown in Figure 8 that, regardless of energy density, removal of oxygen leads instantly to a significant increase in the PL emission of the ZnO nanoparticles. Yet the specific characteristics of the %Δ*I*(*t*) curves are differentiated both in amplitude and temporal profile with varying excitation energy density. At low *F_exc_* (e.g., 0.05 mJ/cm^2^) the emission enhancement is noticeably profound compared to high *F_exc_* values (e.g., 2 mJ/cm^2^), nevertheless this occurs at the expense of the time required to reach equilibrium which is significantly longer. One could alternatively consider that under high *F_exc_* conditions the sensor reaches quickly to saturation (see Figure 8). 

**Figure 9:**
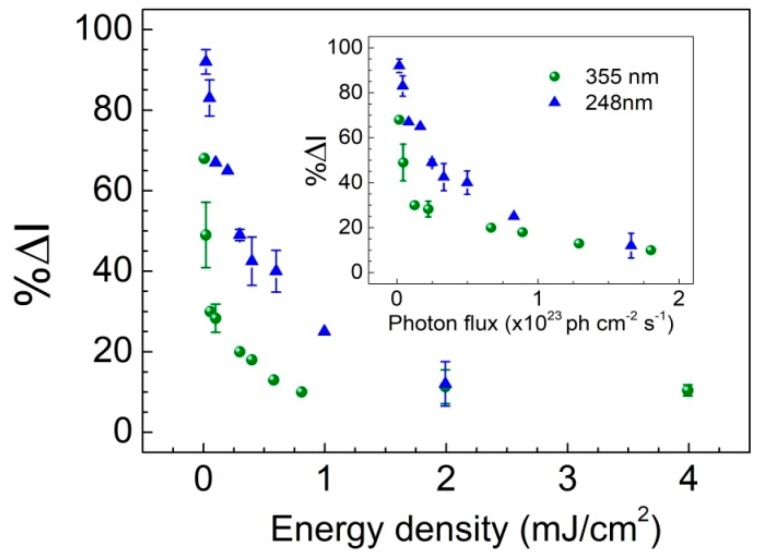
Sensor response, %Δ*I*, as a function of excitation energy density with 248 and 355 nm laser pulses (corresponding to power density values from 10^3^ to 10^5^ Watt/cm^2^) following exposure of ZnO-PDMS nanohybrid to atmospheric air-low vacuum cycles. Inset: %Δ*I* as a function of photon flux.

The global view of the sensor response, observed in the air-to-vacuum transition, is shown in Figure 9, as a function of pump energy density for both 248 nm and 355 nm excitation. 

Similarly, as to the case of ethanol sensing, most efficient performance is recorded for low excitation fluence, while for *F_exc_* > 0.2 mJ/cm^2^ (Mott transition), the system enters the low plateau regime of minimum response. It is, furthermore, very interesting to note that all data points in Figure 9 (see inset) appear to display a very similar behavior when the response dependence is viewed as a function of photon flux instead of energy density.

**Figure 10:**
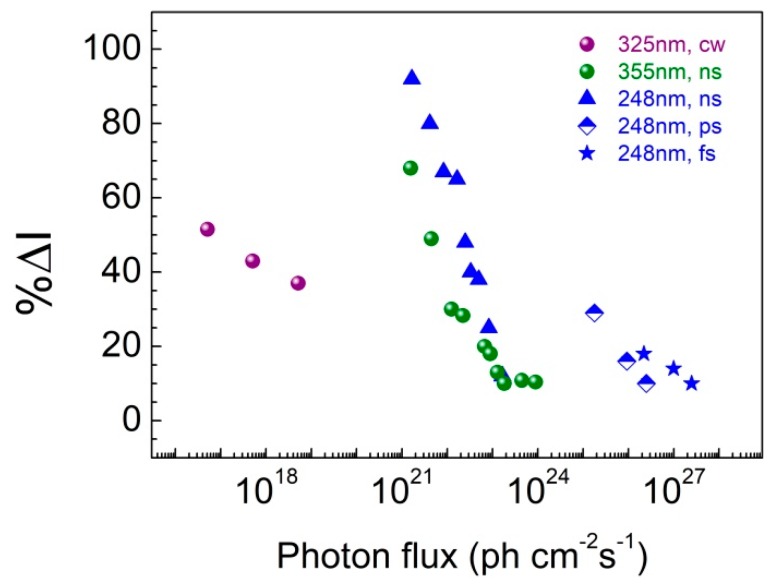
Sensor response, %Δ*I*, as a function of photon flux, following exposure of ZnO-PDMS nanohybrid to atmospheric air-low vacuum cycles, under excitation with laser pulses of different wavelength (325, 248, and 355 nm) and pulse duration (cw, ns, ps, fs).

Finally, in an effort to evaluate the PL-based oxygen sensing response beyond the case of nanosecond pulsed excitation, we investigated briefly two additional sources of excitation: (a) a cw He-Cd laser emitting at *λ_exc_* = 325 nm and (b) a KrF excimer laser emitting 0.5 ps and 5ps pulses at *λ_exc_* = 248 nm [35]. These additional sources expand the power density range investigated across 10 orders of magnitude, ranging from 10^−2^ to 10^9^ Watt/cm^2^ (Figure 10). 

As regards pulsed excitation it becomes evident from the data in Figure 10 that the main factor determining response appears to be the photon flux of the excitation source. The maximum response of ZnO-PDMS hybrid to oxygen in atmospheric air-low vacuum cycles is approaching 100%, significantly higher compared to that reported in the current literature [23,34], and was achieved by use of the nanosecond-pulsed KrF laser. Measurements with pico- or femto-second pulses were performed at an energy density range similar to that for the nanosecond experiments but, given the much narrower laser pulse width, the flux ended up being considerably higher corresponding to the electron-hole plasma regime, hence the observed low response of the sensor. Based on the argument that the sensor response depends on the excitation photon flux, one would expect that cw excitation would feature even higher response in comparison with the nanosecond case. This has not been the case in our experiments and an alternative interpretation might be required. It is noted, however, that a similar PL-based sensing response (%Δ*I* = 50%) has been observed with nanostructured ZnO thin films subjected to atmospheric air-low vacuum cycles, when excitation was provided by a cw Xe lamp as excitation source (*λ_exc_* = 280 nm) [23].

## 4. Conclusions 

The changes in the photoluminescence emission of ZnO-PDMS nanocomposites fabricated from low-cost, commercially available ZnO nanoparticles can be used to effectively monitor environmental changes in ethanol and oxygen atmosphere. Excitation conditions, such as wavelength and particularly energy density, were found to influence the sensor response. The response was found to be similar at 248 nm and 355 nm excitation, with photon flux playing an essential role on the observed sensing performance. Increase in the density of photons influences the performance of the sensor and reduces its response towards ethanol and oxygen detection. This behavior is attributed to the corresponding increase of the charge carrier density upon irradiation that influence the interaction of the analyte with the semiconductor, which is favored when optical excitation is maintained at very low fluence levels. Interestingly, for excitation density at and above the Mott transition the response appears to reach a minimum that is not changing significantly with increasing *F_exc_*.
